# Intracerebral hemorrhage‐induced brain injury in mice: The role of peroxiredoxin 2‐Toll‐like receptor 4 inflammatory axis

**DOI:** 10.1111/cns.14681

**Published:** 2024-03-22

**Authors:** Yang Du, Jinjin Wang, Jia Zhang, Ning Li, Guangshuo Li, Xinmin Liu, Yijun Lin, Dandan Wang, Kaijiang Kang, Liheng Bian, Xingquan Zhao

**Affiliations:** ^1^ Department of Neurology Beijing Tiantan Hospital, Capital Medical University Beijing China; ^2^ China National Clinical Research Center for Neurological Diseases Capital Medical University Beijing China; ^3^ Laboratory for Clinical Medicine Capital Medical University Beijing China; ^4^ Research Unit of Artificial Intelligence in Cerebrovascular Disease Chinese Academy of Medical Sciences Beijing China; ^5^ Center of Stroke, Beijing Institute for Brain Disorders Beijing China

**Keywords:** brain injury, inflammation, intracerebral hemorrhage, mice, peroxiredoxin 2, TLR4

## Abstract

**Background:**

Peroxiredoxin 2 (Prx2), an intracellular protein that regulates redox reactions, released from red blood cells is involved in inflammatory brain injury after intracerebral hemorrhage (ICH). Toll‐like receptor 4 (TLR4) may be crucial in this process. This study investigated the role of the Prx2‐TLR4 inflammatory axis in brain injury following experimental ICH in mice.

**Methods:**

First, C57BL/6 mice received an intracaudate injection of autologous arterial blood or saline and their brains were harvested on day 1 to measure Prx2 levels. Second, mice received an intracaudate injection of either recombinant mouse Prx2 or saline. Third, the mice were co‐injected with autologous arterial blood and conoidin A, a Prx2 inhibitor, or vehicle. Fourth, the mice received a Prx2 injection and were treated with TAK‐242, a TLR4 antagonist, or saline (intraperitoneally). Behavioral tests, magnetic resonance imaging, western blot, immunohistochemistry/immunofluorescence staining, and RNA sequencing (RNA‐seq) were performed.

**Results:**

Brain Prx2 levels were elevated after autologous arterial blood injection. Intracaudate injection of Prx2 caused brain swelling, microglial activation, neutrophil infiltration, neuronal death, and neurological deficits. Co‐injection of conoidin A attenuated autologous arterial blood‐induced brain injury. TLR4 was expressed on the surface of microglia/macrophages and neutrophils and participated in Prx2‐induced inflammation. TAK‐242 treatment attenuated Prx2‐induced inflammation and neurological deficits.

**Conclusions:**

Prx2 can cause brain injury following ICH through the TLR4 pathway, revealing the Prx2‐TLR4 inflammatory axis as a potential therapeutic target.

## Graphical abstract

Brain Prx2 levels are elevated as red blood cell lysis increases after autologous arterial blood injection and increased Prx2 levels can cause inflammatory brain injury through the TLR4 pathway. Additionally, Prx2‐TLR4 inflammatory axis may be a potential therapeutic target for secondary brain injury following ICH.

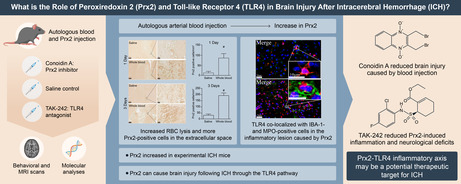

## 1 INTRODUCTION

Spontaneous intracerebral hemorrhage (ICH) accounts for 15‐20% of all strokes and is associated with high morbidity and mortality.^1^ After the onset of ICH, a progressive inflammatory response occurs around the hematoma,^2^, ^3^ ultimately leading to deterioration of neurological function, in which red blood cell (RBC) lysis products play a crucial role.^2^

Peroxiredoxins are a group of proteins that regulate intracellular redox reactions and maintain normal cellular metabolism, including six isoforms (Prx1‐6).^4^ Peroxiredoxin 2 (Prx2) is abundant in RBC cytoplasm at a concentration of 5.6 mg/mL,^5^ third only to hemoglobin and carbonic anhydrase‐1.^6^ When RBCs undergo lysis, Prx2 released outside the cells quickly initiates and induces inflammation in the brain, leading to neurological deficits.^7^, ^8^ However, the mechanism underlying this pathology remains unclear.

Toll‐like receptors (TLRs) are members of a family of pattern‐recognition receptors that detect both exogenous (pathogen‐associated molecular patterns, PAMPs) and endogenous ligands (damage‐associated molecular patterns, DAMPs). Therefore, TLRs play a critical role in the innate immune system by activating inflammatory signaling pathways.^9^, ^10^ A study on subarachnoid hemorrhage suggested that Prx2 interacts with TLR4 on microglia, leading to microglial activation.^11^ This indicates that the TLR4 signaling pathway may be involved in the Prx2‐mediated inflammatory response. However, the roles of the extracellular Prx2 and TLR4 signaling pathways in the inflammatory process after ICH have not been elucidated.

Conoidin A is a mammalian cell‐permeable inhibitor of Prx1 and Prx2.^12^, ^13^ As the inhibitor of Prx2, conoidin A is considered effective in both a rat ICH model^7^ and rat intraventricular hemorrhage model,^14^ and further validation is urgently needed in other species. Furthermore, TAK‐242 (Ethyl (6R)‐6‐[N‐(2‐chloro‐4‐fluorophenyl) sulfamoyl] cyclohex‐1‐ene‐1‐carboxylate), an exogenous synthetic antagonist for TLR4, has been shown to reduce inflammatory injury in a mouse model of ICH.^15^ Therefore, in this study, we aimed to examine (1) the role of Prx2 in the inflammatory process after mouse experimental ICH; (2) whether the TLR4 signaling pathway is involved in the inflammatory process induced by Prx2; and (3) whether conoidin A and TAK‐242 could be potential therapeutic agents to treat the Prx2‐TLR4 inflammatory axis during mouse experimental ICH.

## 2 METHODS

### 2.1 Animal preparation and intracerebral infusion

The protocols for these animal procedures were approved by the Ethics Committee of Beijing Neurosurgery Research Institute. A total of 91 male C57BL/6 mice (8–12 weeks old) were purchased from Charles River Laboratories (Beijing, China). The mice were exposed to a 12‐h light/dark cycle, bred as specified pathogen‐free, and provided food and water ad libitum. ICH modeling was performed as previously reported.^16^ Mice were anesthetized by intraperitoneal injection of tribromoethanol (240 mg/kg, T903147, Macklin, Shanghai, China), and body temperature was maintained at 37°C with a heating pad. Autologous arterial blood was acquired from the right femoral artery, and the mice were placed in a stereotaxic frame (RWD, Shenzhen, China). A 0.6‐mm cranial burr hole was made, and autologous arterial blood (30 μL) was injected at a rate of 3 μL/min using a 26‐gauge needle at the coordinates:0.2 mm anterior, 2.5 mm lateral, and 3.5 mm ventral to the bregma. The needle was maintained at the injection point for 10 min to prevent fluid backflow. The cranial pinhole was closed with bone wax, and the skin was sutured. This study complied with the ARRIVE guidelines (Animal Research: Reporting of In Vivo Experiments) for in vivo experiments. Randomization was performed using odd or even numbers.

The Prx2 inhibitor conoidin A (15,605; Cayman Chemical, Ann Arbor, Michigan, USA) was diluted in dimethyl sulfoxide (DMSO) at a concentration of 5 mmol/L and then co‐injected with autologous arterial blood (1:100 dilution) at a final concentration of 50 μmol/L. The same volume of DMSO was co‐injected with autologous arterial blood (1:100 dilution) as the vehicle control. The TLR4 antagonist TAK‐242 (13,871; Cayman) was dissolved in DMSO and diluted in double‐distilled water. The final DMSO concentration was 1%. Dissolved TAK‐242 (3 mg/kg) or an equal volume of DMSO (1%) was injected intraperitoneally immediately after Prx2 injection. Optimal doses of conoidin A and TAK‐242 were selected based on previous studies.^14^, ^15^, ^17^

### 2.2 Experimental groups

There are four parts in this study. First, mice had an intracerebral injection of arterial blood (30 μL, *n* = 4) or saline (30 μL, *n* = 4) and were euthanized at 24 h after surgery for western blotting analysis. In addition, 20 mice received an intracerebral injection of either blood (30 μL, *n* = 10) or saline (30 μL, *n* = 10), and five mice from each group were euthanized on day 1, while the other five mice from each group were euthanized on day 3 after injection. The brains were then prepared for histological examination.

Second, mice received an intracerebral injection of either recombinant mouse Prx2 (NBP2‐61185; Novus Biologicals, Centennial, Colorado, USA) (10 μL, *n* = 14) or saline (10 μL, *n* = 12). All the mice underwent behavioral testing and magnetic resonance imaging (MRI) at 24 h after injection. The mice were euthanized for brain histology (*n* = 7 for the Prx2 group and *n* = 7 for the saline group) and RNA‐seq (*n* = 7 for the Prx2 group and *n* = 5 for the saline group).

Third, 16 mice were randomly divided into two groups and had a 30‐μL intracerebral injection of arterial blood + conoidin A or arterial blood + vehicle (DMSO). All mice had MRI scans and behavioral testing after 3 days. Finally, the mice were euthanized for brain histology.

Fourth, 16 mice received an intracerebral injection of recombinant mouse Prx2 (10 μL). They were then evenly divided into a TAK‐242 group and a control group and treated with an intraperitoneal injection of dissolved TAK‐242 (3 mg/kg) or an equal volume of DMSO (1%) immediately after the intracerebral injection. Mice were euthanized on day 1 after the MRI scans and behavioral testing. The brains were then used for histological analyses. Animals that had died were excluded from the study.

### 2.3 Magnetic resonance imaging and brain swelling measurement

MRI was performed using a 7.0‐T MRI scanner (BioClinScan Animal MRI System; Bruker, Karlsruhe, Germany) at separate time points (details are provided in the preceding experimental protocols). Apparent diffusion coefficients (ADC), T2 values, and susceptibility‐weighted imaging (SWI) were obtained. All MRI images were measured using NIH ImageJ software. Brain swelling was calculated using MRI as follows: volume of ([ipsilateral hemisphere‐contralateral hemisphere]/volume of contralateral hemisphere) × 100%.^18^

### 2.4 Western blot analysis

Mice were anesthetized and their brains were perfused with phosphate‐buffered saline (PBS; 0.1 M). The brains were then removed, and a 3‐mm‐thick coronal slice centered on the injection site was cut. The slices were separated into the ipsilateral and contralateral basal ganglia. Protein lysates were prepared from brain tissue. Briefly, these protein lysates were separated by sodium dodecyl sulfate‐polyacrylamide gel electrophoresis and transferred onto hydrophobic PVDF transfer membrane (ISEQ00010, 0.2 μm pore size; MilliporeSigma, Rockville, Maryland, USA). The PVDF membranes were incubated with the primary antibodies including rabbit anti‐Prx2 (NBP2‐67887, 1:1000; Novus Biologicals, Centennial, Colorado, USA) and mouse anti‐β‐actin (TA346894, 1:5000; Origene, Beijing, China), followed by incubation with peroxidase‐conjugated secondary antibodies (31,460, 1:5000; ThermoFisher Scientific, Waltham, Massachusetts, USA). The signals were detected with an ECL system (Amersham ImageQuant 800, GE Healthcare, Boston, Massachusetts, USA). The relative densities of bands against β‐actin were analyzed with NIH ImageJ software.

### 2.5 RNA‐seq and analysis

Total RNA was extracted from the lesion region of the injection hemisphere using TRIzol reagent (15,596,026; Invitrogen, ThermoFisher Scientific). Contaminating DNA was removed by digesting the remnants with DNase I (18,047,019; Invitrogen, ThermoFisher Scientific). Quality control for RNA concentration and integrity was performed using an Agilent 2100 Bioanalyzer (Santa Clara, California, USA). RNA samples were reverse‐transcribed with Oligo T primers to produce cDNA, and dsDNA samples were generated using dNTPs, DNA polymerase, and RNase H. The dsDNA was then purified by magnetic separation, and the Tn5 marker was carried out. The library was prepared using a TruePrep DNA Library Prep Kit V2 (cat. TD503‐1; Vazyme, Nanjing, China). All samples were sequenced on Illumina HiSeq 2000 platform (San Diego, California, USA) with 150 bp paired‐end reads. Mean fragments per kilobase of transcript per million mapped reads (FPKM) were utilized to screen the differential genes, which applied for differential gene ontology (GO) classification and Kyoto Encyclopedia of Genes and Genomes (KEGG) enrichment analysis.^19^

### 2.6 Immunohistochemistry and immunofluorescence staining

Mice were anesthetized and subjected to transcardial perfusion with 4% paraformaldehyde. Paraffin‐embedded brains were sliced into 8‐μm sections. The primary antibodies used for immunohistochemical staining and immunofluorescence staining were rabbit anti‐Prx2 IgG (10545‐2‐AP, 1:200; Proteintech, Wuhan, China), rabbit anti‐IBA‐1 (ionized calcium‐binding adaptor molecule 1, 10904‐1‐AP, 1:400; Proteintech), rabbit anti‐MPO IgG (myeloperoxidase, PA5‐16672, 1:400; ThermoFisher Scientific), and goat anti‐MyD88 (myeloid differentiation primary response protein 88, EB06667, 1:200; Everest Biotech, Oxford, UK). The secondary antibodies used were goat anti‐rabbit IgG H&L (Cy5®) preadsorbed (ab6564, 1:1000; Abcam, Cambridge, UK) and donkey anti‐goat IgG H&L (Alexa Fluor® 594) (ab150132, 1:400; Abcam). Negative controls were treated similarly but omitted the primary antibody.

The immunofluorescence double staining was based on tyramide signal amplification (TSA),^20^ and the primary antibodies were rabbit anti‐IBA‐1 (10904‐1‐AP, 1:400; Proteintech), rabbit anti‐MPO IgG (PA5‐16672, 1:400; ThermoFisher Scientific), and rabbit anti‐TLR4 IgG (19811‐1‐AP, 1:200; Proteintech). The secondary antibodies used were HRP‐conjugated goat anti‐rabbit IgG (H + L) (GB23303, 1:500; Servicebio, Wuhan, China) and Cy3 conjugated goat anti‐rabbit IgG (H + L) (GB21303, 1:300; Servicebio). The TSA used were iF488‐Tyramide (G1231, 1:500; Servicebio) and iF647‐Tyramide (G1232, 1:400; Servicebio). Nuclei were labeled with DAPI (F6057; Sigma‐Aldrich, St. Louis, Missouri, USA).^21^

### 2.7 Fluoro‐Jade C staining

To assess neuronal degeneration, the mouse brain sections were stained with Fluoro‐Jade C (AG325; MilliporeSigma).^6^

### 2.8 Cell counting

Cell counting was performed on the coronal brain sections by blinded observers. A digital camera was used to capture three high‐power images (40× magnification) of the different brain areas. All measurements were repeated thrice, and the mean values were used.^16^

### 2.9 Behavioral tests

Neurological deficits were assessed using corner turn tests and forelimb use asymmetry in all animals as previously described.^7^ Behavioral tests were performed by a blinded observer.

### 2.10 Statistical analysis

Statistical analysis was performed using GraphPad Prism software. The presence of a normal distribution was determined using the Kolmogorov‐Smirnov test. Unpaired Student's *t*‐test and one‐way ANOVA with Tukey's multiple comparisons test were used for data with a normal distribution and values are displayed as means ± standard deviation (SD). Mann‐Whitney U‐tests and nonparametric Kruskal–Wallis test with Dunn's multiple comparisons test were used for data with a nonnormal distribution and values are displayed as median (25th percentile, 75th percentile). Significant differences were considered as *p* < 0.05.^7^

## 3 RESULTS

The mortality of the mice in this study was 5% (5/91); all mice that had died were excluded from the analyses because they failed to complete the entire course of the experiment.

**FIGURE 1:**
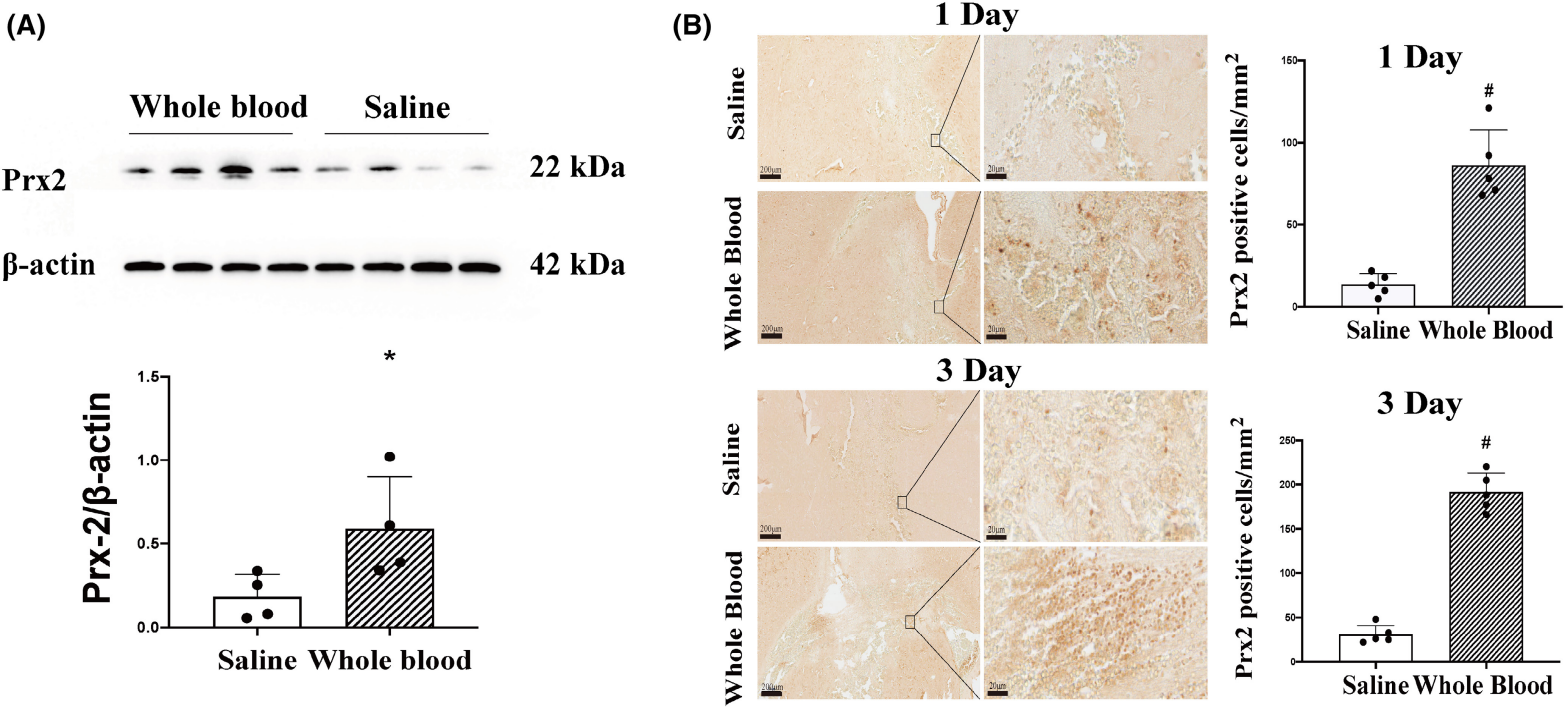
Intracaudate injection of autologous arterial blood increases brain Prx2 (peroxiredoxin 2) levels. (A) Western blot measuring Prx2 levels in the ipsilateral basal ganglia at 24 h after autologous arterial blood or saline injection (30 μL). Values are mean ± SD; *n* = 4 for both groups; *p < 0.05 vs. saline group. (B) The numbers of Prx2‐positive cells were quantified through immunohistochemistry on days 1 and 3 after autologous arterial blood injection. Values are mean ± SD; *n* = 5 for all groups; #*p* < 0.01 versus saline group. Scale bar = 200 μm at low magnification, 20 μm at high magnification.

Prx2 protein levels were assessed using western blotting. Compared to the saline group, the Prx2 protein level in the ipsilateral basal ganglia significantly increased 24 h after injection of 30‐μL autologous arterial blood (Prx2/β‐actin, 0.59 ± 0.31 vs. 0.18 ± 0.14 in the saline group; *p* < 0.05, Figure 1A). In addition, compared to the saline group, the number of Prx2‐positive cells significantly increased on both day 1 (86 ± 22 vs. 14 ± 7 cells/mm^2^ in the saline group; *p* < 0.01; Figure 1B) and day 3 (191 ± 22 vs. 31 ± 10 cells/mm^2^ in the saline group; *p* < 0.01; Figure 1B) after injection of 30‐μL autologous arterial blood.

**FIGURE 2:**
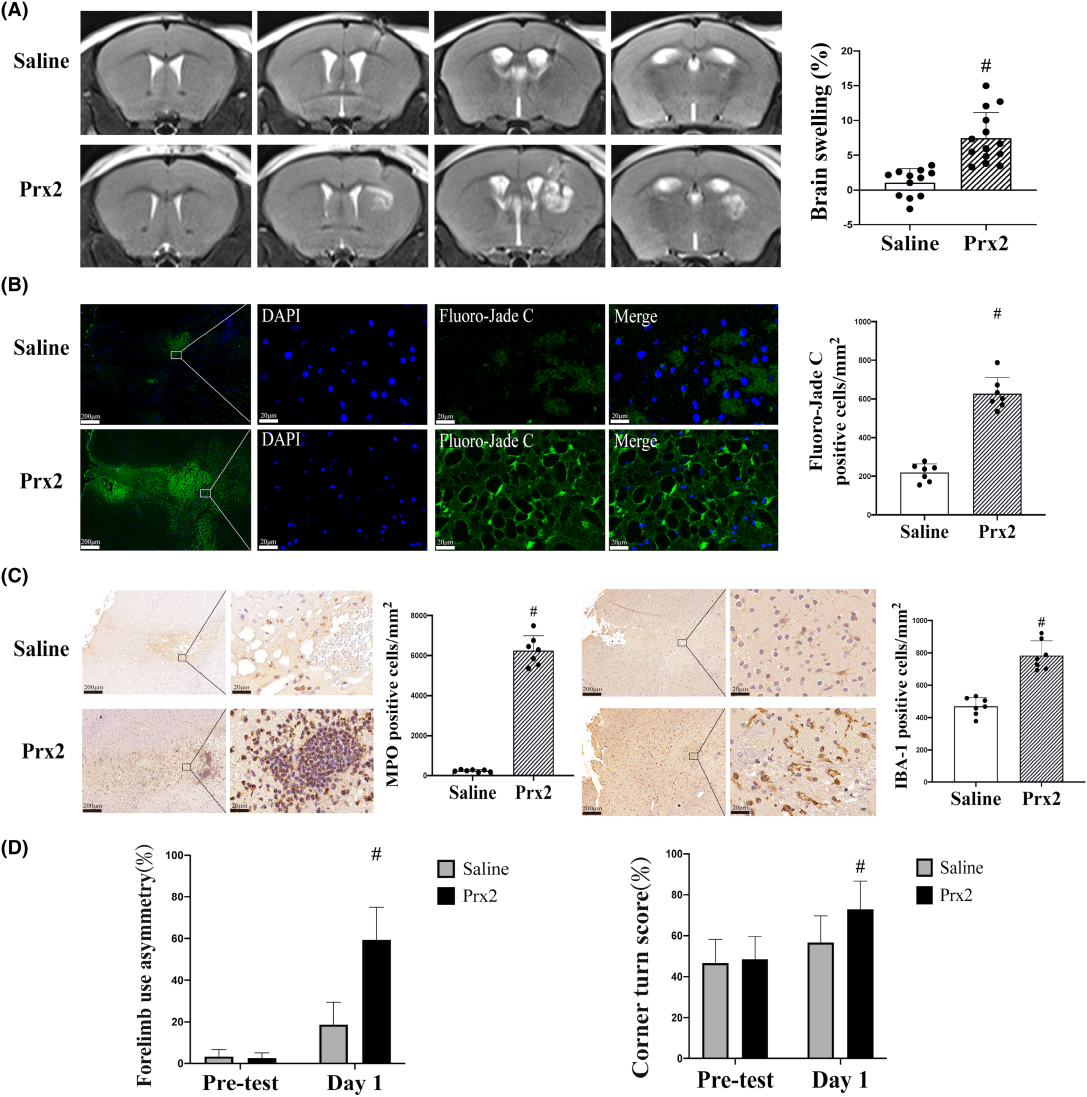
Intracaudate injection of Prx2 (peroxiredoxin 2) induces brain injury and neurological deficits. (A) T2 MRI evaluation of brain swelling at 24 h after the injection of saline or Prx2 (10 μL). Values are mean ± SD; *n* = 12 for saline and *n* = 14 for Prx2; #*p* < 0.01 versus saline group. (B) Fluoro‐Jade C positive cells in the ipsilateral basal ganglia at 24 h after the injection of saline or Prx2. Values are mean ± SD; *n* = 7 for all groups; #*p* < 0.01 versus saline group. Scale bar = 200 μm at low magnification, 20 μm at high magnification. (C) MPO (myeloperoxidase) and IBA‐1 (ionized calcium‐binding adaptor molecule 1) immunohistochemistry at the ipsilateral basal ganglia 24 h after saline and Prx2 injection. Values are mean ± SD; *n* = 7 for all groups; #*p* < 0.01 versus saline group. Scale bar = 200 μm at low magnification, 20 μm at high magnification. (D) Forelimb use asymmetry and corner turn tests before and 1 day after saline or Prx2 injection. Values are mean ± SD; *n* = 12 for saline, *n* = 14 for Prx2; #*p* < 0.01 versus saline group on both tests.

T2‐weighted MRI was performed to examine brain swelling on day 1 after injection of 10‐μL recombinant mouse Prx2 or saline into the right basal ganglia. Prx2 injection resulted in significant brain swelling in the ipsilateral hemisphere (7.4 ± 3.70%) compared with saline injection (1.1 ± 1.99%; *p* < 0.01; Figure 2A). In addition, there were more Fluoro‐Jade C‐positive cells in the Prx2 group compared with controls (627 ± 83 vs. 220 ± 46 cells/mm^2^ in the saline control, *p* < 0.01; Figure 2B). Prx2 injection caused a significant increase in the number of MPO‐positive cells (6243 ± 741 vs. 236 ± 57 cells/mm^2^ in the saline group; *p* < 0.01; Figure 2C) and IBA‐1‐positive cells (783 ± 90 vs. 469 ± 55 cells/mm^2^ in the saline group; *p* < 0.01, Figure 2C) in the ipsilateral basal ganglia compared with controls. To measure functional outcomes, we conducted forelimb use asymmetry and corner turn tests. Prx2‐injected mice had more behavioral deficits than saline‐injected mice (forelimb use asymmetry, *p* < 0.01, Figure 2D; corner turn, *p* < 0.01, Figure 2D).

**FIGURE 3:**
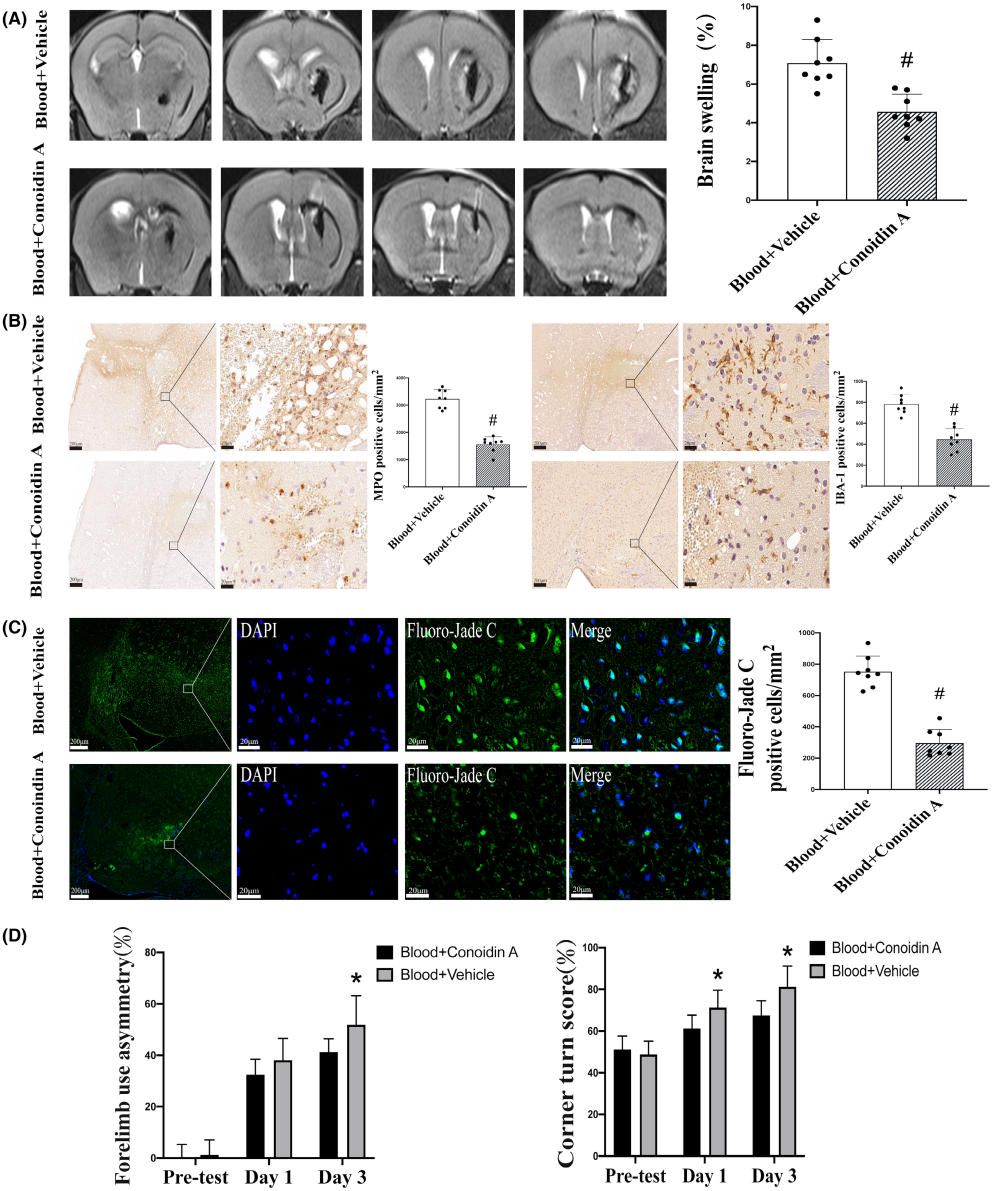
The effect of conoidin A on autologous arterial blood‐induced brain injury and neurological deficits. (A) T2 MRI evaluation of brain swelling on day 3 after the co‐injection of vehicle or conoidin A with autologous arterial blood. Values are mean ± SD; *n* = 8 for both groups; #*p* < 0.01 versus vehicle group. (B) MPO (myeloperoxidase) and IBA‐1 (ionized calcium‐binding adaptor molecule 1) immunohistochemistry at the ipsilateral basal ganglia 3 days after the co‐injection of vehicle or conoidin A with autologous arterial blood. Values are mean ± SD; *n* = 8 for both groups; #*p* < 0.01 versus saline group. Scale bar = 200 μm at low magnification, 20 μm at high magnification. (C) Fluoro‐Jade C‐positive cells in the ipsilateral basal ganglia at 3 days after the co‐injection of vehicle or conoidin A with autologous arterial blood. Values are mean ± SD; *n* = 8 for both groups; #*p* < 0.01 versus vehicle group. Scale bar = 200 μm at low magnification, 20 μm at high magnification. (D) Forelimb use asymmetry and corner turn tests before and 3 days after the co‐injection of vehicle or conoidin A with autologous arterial blood. Values are mean ± SD; *n* = 8 for both groups; *p < 0.05 versus vehicle group at day 1 and day 3.

Co‐injection of a Prx2 inhibitor, conoidin A, significantly reduced the brain swelling induced by autologous arterial blood (7.1 ± 1.22%) compared with the vehicle co‐injection group (4.6 ± 0.90%) after 3 days (*p* < 0.01; Figure 3A). Conoidin A co‐injection also attenuated autologous arterial blood‐induced neutrophil infiltration and microglia/macrophage activation. Compared with the control group, there was a significant decrease in the number of MPO‐positive cells (1560 ± 276 vs. 3228 ± 341 cells/mm^2^ with vehicle co‐injection, *p* < 0.01; Figure 3B) and IBA‐1‐positive cells (445 ± 105 vs. 782 ± 91 cells/mm^2^ with vehicle co‐injection, *p* < 0.01; Figure 3B) in mice where autologous arterial blood was co‐injected with conoidin A. Conoidin A co‐injection also attenuated autologous arterial blood induced neuronal degeneration as assessed by Fluoro‐Jade C staining (296 ± 85 vs. 753 ± 99 cells/mm^2^; *p* < 0.01; Figure 3C). In addition, conoidin A co‐injection alleviated behavioral deficits induced by autologous arterial blood. On day 3 after injection, forelimb placing scores in the conoidin A co‐injected group were better than those in the vehicle group (41.3 ± 5.2% vs. 51.9 ± 11.3% in vehicle group; *p* < 0.05; Figure 3D), although the difference was not significant on day 1. The corner turn test results of the conoidin A co‐injected group showed significant differences on both day 1 (*p* < 0.05; Figure 3D) and day 3 (*p* < 0.05; Figure 3D).

**FIGURE 4:**
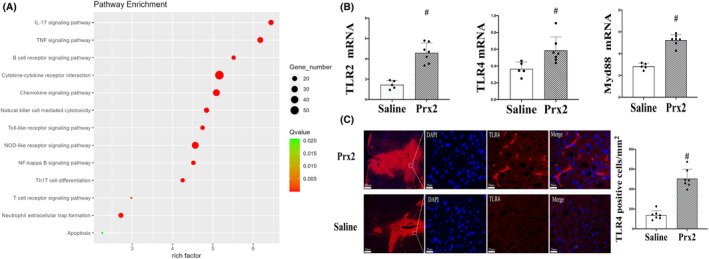
TLR (toll‐like receptor) signaling pathway is upregulated in the inflammation induced by Prx2 (peroxiredoxin 2). (A) KEGG (Kyoto Encyclopedia of Genes and Genomes) pathway analysis of DEGs (differentially expressed genes) in the ipsilateral basal ganglia at 24 h after saline or Prx2 injection (10 μL); *n* = 5 for saline and *n* = 7 for Prx2. Dot color represents Q‐value from the least significant (green) to the most significant (red); dot size represents gene number, the number of significant DEGs in the KEGG pathway; rich factor represents the fraction of significant DEGs among all genes in the KEGG pathway. (B) FPKM (Mean fragments per kilobase of transcript per million mapped reads) values of TLR2, TLR4, Myd88 (myeloid differentiation primary response protein 88) in the ipsilateral basal ganglia at 24 h after saline or Prx2 injection. Values are mean ± SD; *n* = 5 for saline and *n* = 7 for Prx2; #*p* < 0.01 versus saline group. (C) TLR4 immunofluorescence at the ipsilateral basal ganglia 24 h after saline or Prx2 injection. Values are mean ± SD; *n* = 7 for both groups; #*p* < 0.01 versus saline group. Scale bar = 200 μm at low magnification, 20 μm at high magnification.

We used RNA‐seq to analyze inflammatory brain injury caused by Prx2 and conducted a KEGG enrichment analysis. Then, we identified 13 upregulated pathways, one of which is the TLR signaling pathway (Figure 4A). In addition, compared to the saline group, the FPKM values of TLR2 (4.59 ± 0.97 vs. 1.46 ± 0.41 in the saline group; *p* < 0.01; Figure 4B), TLR4 (0.59 ± 0.16 vs. 0.37 ± 0.08 in the saline group; *p* < 0.01; Figure 4B), and MyD88 (5.24 ± 0.51 vs. 2.84 ± 0.29 in the saline group; *p* < 0.01; Figure 4B) significantly increased in the Prx2 injection group. Histological analysis showed a significant increase in TLR4‐positive cells (505 ± 95 vs. 138 ± 45 cells/mm^2^ in the saline group; *p* < 0.01, Figure 4C) at the ipsilateral basal ganglia 24 h after Prx2 injection compared with the control group.

**FIGURE 5:**
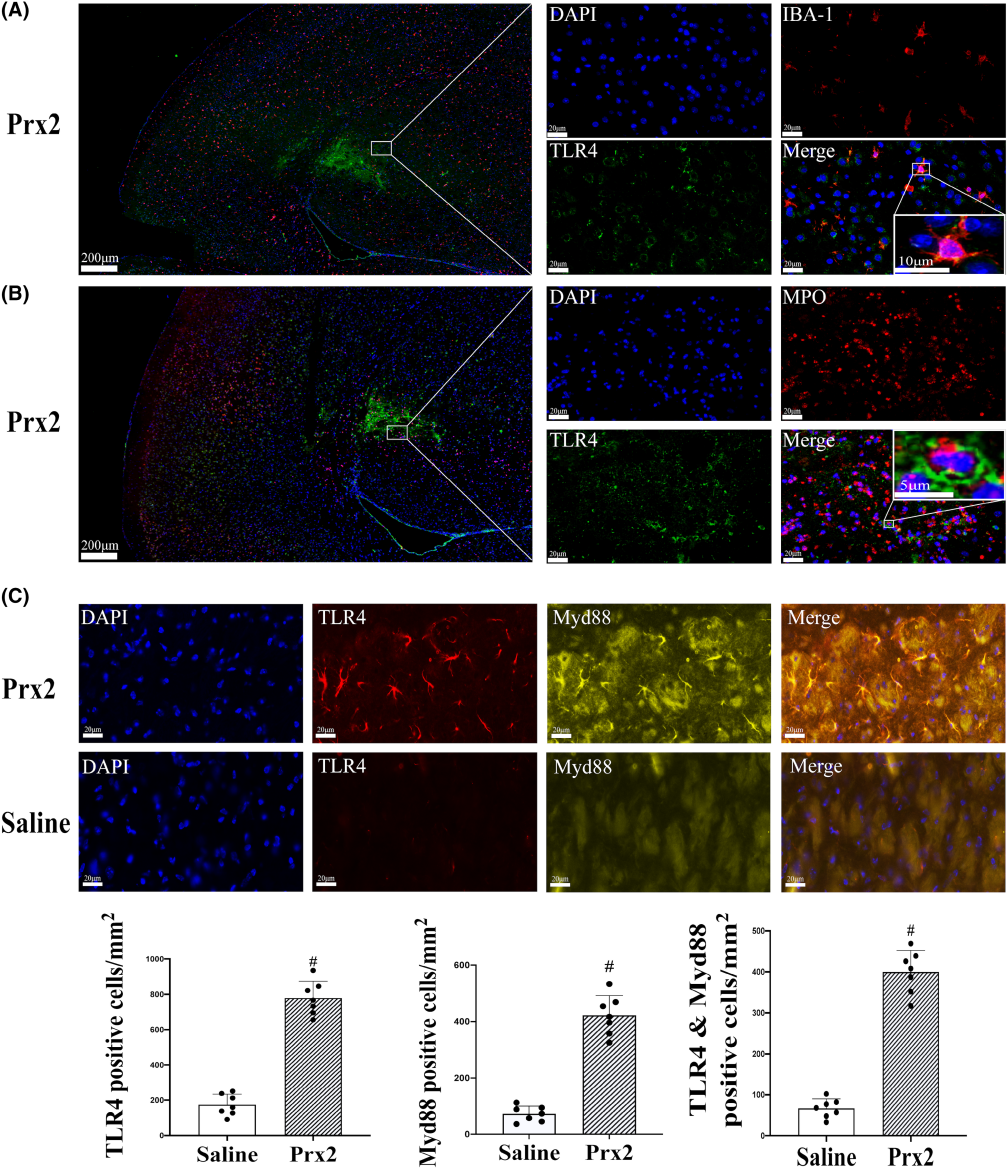
Intracaudate injection of Prx2 (peroxiredoxin 2) activates TLR4 (toll‐like receptor 4) signaling pathway. (A) Triple immunofluorescence labeling of IBA‐1 (ionized calcium‐binding adaptor molecule 1) and TLR4 at the ipsilateral basal ganglia 24 h after Prx2 injection. Co‐localization of TLR4 with IBA‐1 (microglia/macrophage marker). Scale bar = 200 μm at low magnification, 20 μm at high magnification, 10 μm at the highest magnification. (B) Triple immunofluorescence labeling of MPO (myeloperoxidase) and TLR4 at the ipsilateral basal ganglia 24 h after Prx2 injection. Co‐localization of TLR4 with MPO (neutrophil marker). Scale bar = 200 μm at low magnification, 20 μm at high magnification, 5 μm at the highest magnification. (C) Triple immunofluorescence labeling of TLR4 and Myd88 (myeloid differentiation primary response protein 88) at the ipsilateral basal ganglia 24 h after saline or Prx2 injection. Values are mean ± SD; *n* = 7 for both groups; #*p* < 0.01 versus saline group. Scale bar = 200 μm at low magnification, 20 μm at high magnification.

TLR4‐positive cells co‐localized with IBA‐1‐ and MPO‐positive cells. The TLR4‐positive cells were microglia/macrophages (Figure 5A) and neutrophils (Figure 5B) in the lesion center and the basal ganglia around the injection center. After 24 h, compared with the saline group, triple immunofluorescence showed a significant increase in the number of TLR4‐positive cells following Prx2 injection (779 ± 96 vs. 174 ± 61 cells/mm^2^ in the saline control, *p* < 0.01; Figure 5C), MyD88‐positive cells (422 ± 70 vs. 72 ± 28 cells/mm^2^ in the saline control, *p* < 0.01; Figure 5C), and positive cells co‐located with TLR4 and MyD88 (399 ± 52 vs. 67 ± 23 cells/mm^2^ in the saline control, *p* < 0.01; Figure 5C).

**FIGURE 6:**
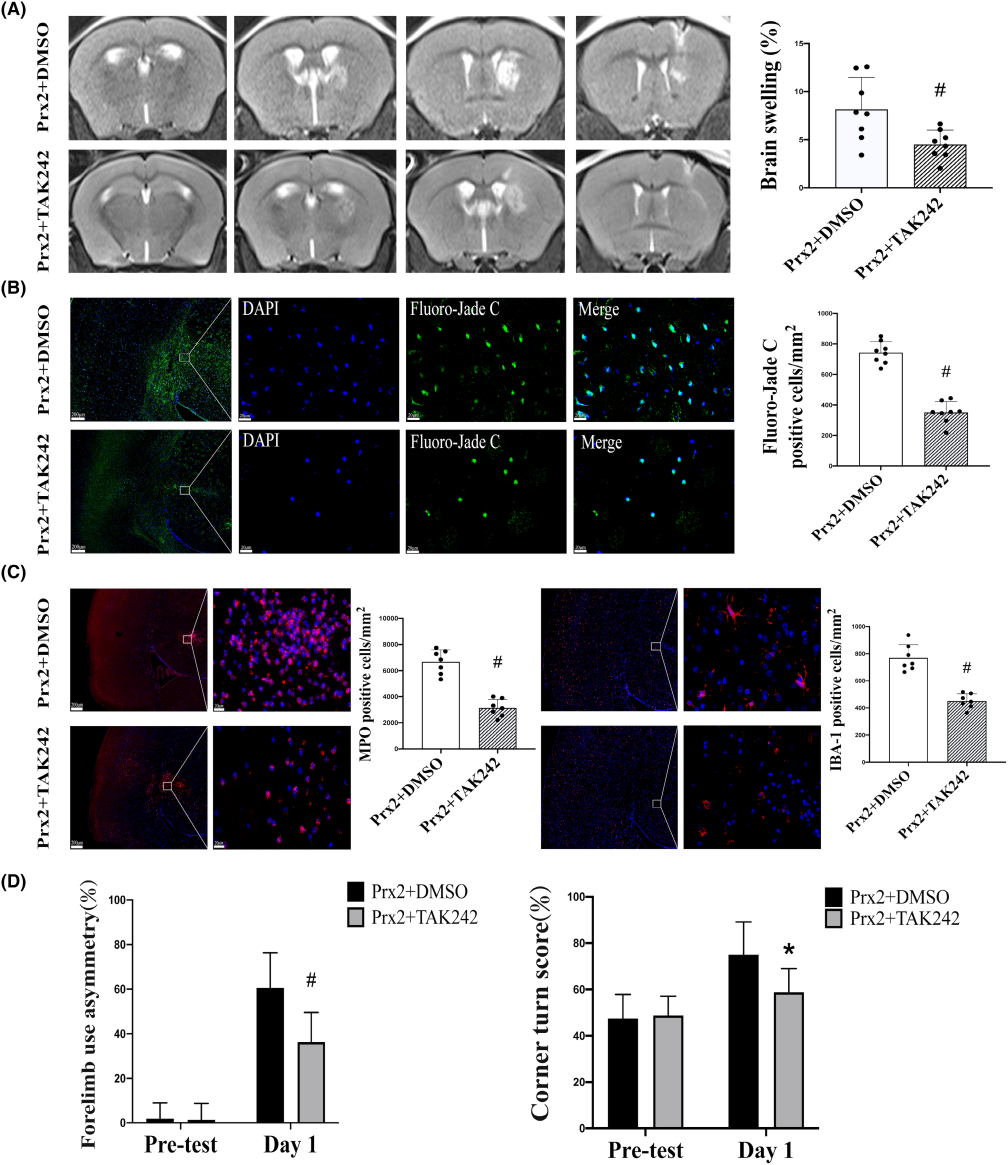
The effect of TAK‐242 on Prx2 (peroxiredoxin 2) induced brain injury and neurological deficits. (A) T2 MRI evaluation of brain swelling at 24 h after the injection of Prx2 with or without TAK‐242 administration. Values are mean ± SD; *n* = 8 for both groups; #*p* < 0.01 versus vehicle group. (B) Fluoro‐Jade C‐positive cells in the ipsilateral basal ganglia at 24 h after the injection of Prx2 with or without TAK‐242 administration. Values are mean ± SD; *n* = 8 for both groups; #*p* < 0.01 versus vehicle group. Scale bar = 200 μm at low magnification, 20 μm at high magnification. (C) MPO (myeloperoxidase) and IBA‐1 (ionized calcium‐binding adaptor molecule 1) immunofluorescence at the ipsilateral basal ganglia 24 h after the injection of Prx2 with or without TAK‐242 administration. Values are mean ± SD; *n* = 8 for both groups; #*p* < 0.01 versus vehicle group. Scale bar = 200 μm at low magnification, 20 μm at high magnification. (D) Forelimb use asymmetry and corner turn tests before and 1 day after the Prx2 injection with or without TAK‐242 administration. Values are mean ± SD; *n* = 8 for both groups; #*p* < 0.01 and *p < 0.05 versus DMSO group at day 1.

Intraperitoneally administered TAK‐242, a TLR4 inhibitor, significantly reduced the brain swelling induced by Prx2 (4.5 ± 1.50%) compared with the control group (8.2 ± 3.31%) at 24 h (*p* < 0.01; Figure 6A). TAK‐242 administration attenuated Prx2‐induced neuronal degeneration as assessed by Fluoro‐Jade C staining (350 ± 71 vs. 743 ± 72 cells/mm^2^; *p* < 0.01; Figure 6B). The number of MPO‐positive cells (3133 ± 662 vs. 6671 ± 918 cells/mm^2^ in the control group, *p* < 0.01; Figure 6C) and IBA‐1 positive cells (450 ± 54 vs. 769 ± 98 cells/mm^2^ in the control group, *p* < 0.01; Figure 6C) significantly decreased in the TAK‐242 administration group. In addition, TAK‐242 administration also alleviated the behavioral deficits induced by Prx2. Forelimb placing scores in TAK‐242 administration group were better than those in the control group (*p* < 0.01; Figure 6D), as were the corner turn tests (*p* < 0.05; Figure 6D).

## 4 DISCUSSION

The major findings of the current study were as follows: (1) injecting autologous arterial blood into the basal ganglia can cause an increase in Prx2 protein levels in the basal ganglia of mouse; (2) intracaudate injection of Prx2 can induce brain swelling, inflammation, neuronal degeneration, and neurological deficits; (3) co‐injection of the Prx2 inhibitor conoidin A attenuated autologous arterial blood‐induced brain swelling, inflammatory responses, neuronal death, and neurological deficits; (4) the TLR signaling pathway was involved in Prx2‐induced inflammation in the basal ganglia and TLR4 was expressed on microglia/macrophage and neutrophils around the injection center; and (5) TAK‐242 treatment (a TLR4 inhibitor) attenuated Prx2‐induced brain swelling, neuronal degeneration, inflammation, and neurological deficits.

Previous studies have shown that intracerebral injection of lysed RBCs can lead to significant brain edema.^22^ As a product of RBC lysis, Prx2 plays an important role in the development of brain edema and inflammation.^7^ Unlike previous studies, the current research simulated ICH by injecting autologous arterial blood into the basal ganglia of mice instead of lysed RBCs, and for the first time, an increase in Prx2 was found in experimental ICH in mice. It is worth noting that over time, RBC lysis increased, and more Prx2 was exposed outside the cell. Therefore, more Prx2‐positive cells can be seen in immunohistochemical staining. Naturally, in subsequent experiments, we validated the significant proinflammatory effects of Prx2. The above findings further elucidate the important role of Prx2 in the inflammatory process of ICH.

Administering the Prx2 inhibitor conoidin A while injecting whole blood into the brain alleviated a series of inflammatory reactions such as brain swelling, which is consistent with previous research.^7^ However, in this study, whole blood was injected into mice further validated the role of conoidin A for ICH in more species, filling a gap in previous research, which may provide more evidence for translational medicine. In addition, this also indirectly confirms the important role of Prx2 in secondary brain injury after ICH.

Prx2 leads to neuroinflammation and brain damage; however, the underlying mechanism is still not fully understood. Related studies suggest that extracellular Prx2, as a member of DAMPs, may activate microglia/macrophages and polymorphonuclear cells via TLR signaling pathways to produce various pro‐inflammatory cytokines and trigger destructive inflammatory responses.^7^, ^11^ TLRs are widely expressed in many types of cells in the human body. In the central nervous system, TLRs are expressed in neurons and immune cells, such as microglia and astrocytes.^23^ Additional evidence suggests that TLR2 and TLR4 are upregulated in microglia and participate in the inflammatory response.^24^, ^25^, ^26^ The RNA‐seq results of this study showed that after injection of Prx2, the mRNA levels of TLR2, TLR4, and MyD88 were significantly upregulated, and the TLR signaling pathways were upregulated according to KEGG analysis, indicating that the TLR pathway was involved in the process of inflammation induced by Prx2. The significant increase in the number of TLR4‐positive cells in the immunohistochemical results further supports this viewpoint.

It is worth noting that in this study, TLR4 co‐localized with IBA‐1‐ and MPO‐positive cells in the inflammatory lesion caused by Prx2, and the inflammation exhibited significant microglia/macrophage activation and neutrophil infiltration. These findings indicated that TLR4 may be expressed on the surface of both microglia/macrophages and neutrophils, and these immune cells from both the brain and circulation may be an essential basis for the excessive inflammatory response seen in ICH. Current research suggests that TLR4 not only mediates the activation of resident immune cells in the brain but also exists on the surface of leukocytes, making it crucial for the infiltration and activation of neutrophils and monocytes.^27^ Studies have shown that TLR4 on the surface of neutrophils can recognize DAMPs released by damaged liver cells, leading to the formation of neutrophil extracellular traps that exacerbate inflammatory damage.^28^ TLR4 also affects CCR2‐mediated immune cell chemotaxis in circulating white blood cells, thereby affecting inflammation.^29^, ^30^ Therefore, TLR4 may be crucial for recognition of Prx2 by both microglia/macrophages and neutrophils.

Once activated, dimerized TLR4 recruits a specific set of adaptor molecules that harbor the toll/IR‐1 (TIR) domain, such as MyD88 and TIR‐domain‐containing adaptor protein‐inducing interferon‐beta (TRIF), and initiate downstream signaling events including activation of the transcription factor nuclear factor kappa B (NF‐kB), leading to the expression of genes encoding inflammation‐associated molecules and cytokines.^26^, ^31^, ^32^ In this study, TLR4 and MyD88 mRNA were upregulated, and triple immunofluorescence showed an increase in the number of co‐localization‐positive cells between TLR4 and MyD88, indicating activation of the TLR4 signaling pathway.

TAK‐242 is a specific antagonist of TLR4 that binds to Cys747 in the intracellular domain, thereby inhibiting the TLR4 signaling pathway.^15^, ^33^ Previous study has shown the neuroprotective effect of TAK242 in experimental ICH.^15^ Consistent with this, our current results further show that TAK‐242 can alleviate brain edema, neuronal degeneration, immune cell activation, and neurological deficits caused by Prx2, emphasizing the role of the Prx2‐TLR4 inflammatory axis in secondary brain injury of ICH. These data also provided more evidence that TAK‐242 may be a promising drug candidate for ICH.

The current study aimed to demonstrate the role of the Prx2 and TLR4 signaling pathways in brain injury after ICH and further explore potential intervention targets. However, this study has several limitations: (1) sex differences were not examined, (2) only one dose and one time point of TAK‐242 administration were tested, and (3) the administration of conoidin A in combination with TAK‐242 on the Prx2‐TLR4 inflammatory axis may require further exploration.

## 5 CONCLUSION

In conclusion, brain Prx2 levels are elevated as red blood cell lysis increases after autologous arterial blood injection and increased Prx2 levels can cause inflammatory brain injury through the TLR4 pathway. Moreover, the Prx2‐TLR4 inflammatory axis may be a potential therapeutic target for secondary brain injury following ICH.

## AUTHOR CONTRIBUTIONS

YD, LHB, and XQZ were responsible for conceptualizing the project and designing. YD, JJW, and XML were responsible for executing, analyzing the results from, drafting, and editing the manuscript. DDW and JZ were responsible for ensuring the validity of the experiments. KJK and GSL were responsible for RNA‐seq data analyses. NL and YJL were responsible for immunofluorescence imaging and image analysis. The authors read and approved the manuscript.

## FUNDING INFORMATION

This study was supported by the National Natural Science Foundation of China (Grant No. 82371302), the Chinese Academy of Medical Sciences Innovation Fund for Medical Sciences (2019‐I2M‐5‐029), and the National Natural Science Foundation of China (Grant No. 82001239).

## CONFLICT OF INTEREST STATEMENT

The authors declare that they have no competing interests.

## Supporting information


Data S1.

## Data Availability

All data generated or analyzed during this study are included in this published article.
