# Supplementary figures and images for: Intracerebral hemorrhage‐induced brain injury in mice: The role of peroxiredoxin 2‐Toll‐like receptor 4 inflammatory axis
Source: CNS Neurosci Ther. 2024 Mar 22;30(3):e14681. doi: 10.1111/cns.14681 (PMC10958402; doi:10.1111/cns.14681)

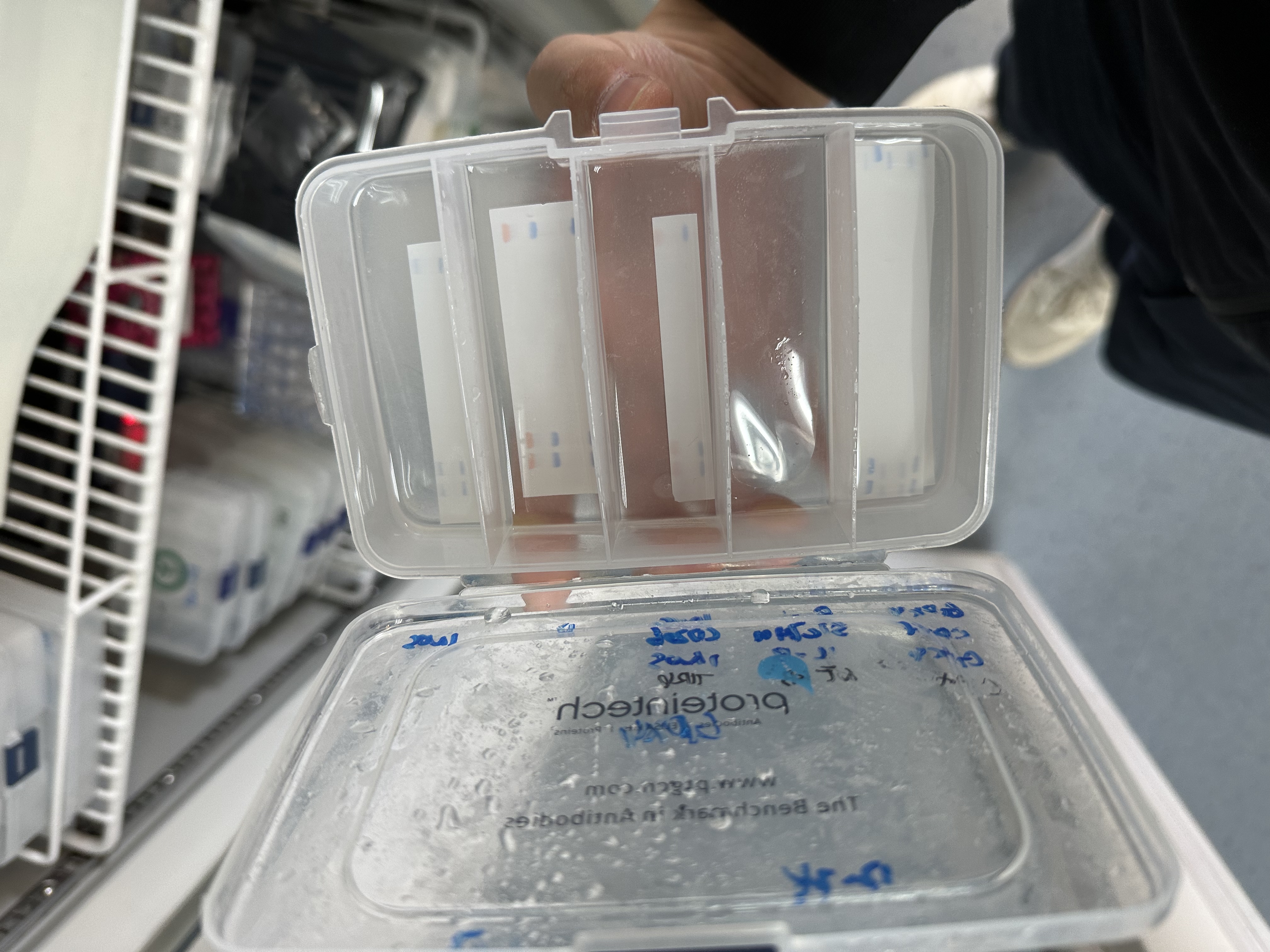

Supplement: Supplementary file 1 — Data S1. [file CNS-30-e14681-s001.zip › Antibody incubating box.jpeg]

25kDa  
20kDa

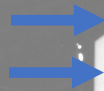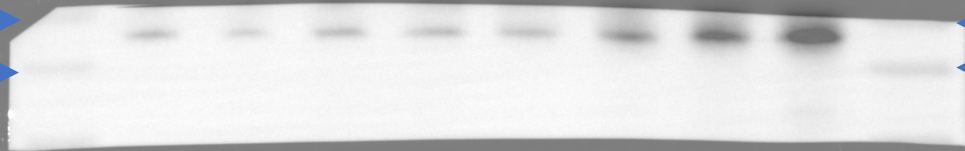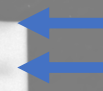

25kDa  
20kDa

Supplement: Supplementary file 1 — Data S1. [file CNS-30-e14681-s001.zip › unedited another prx2 blot.pdf]

40kDa

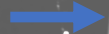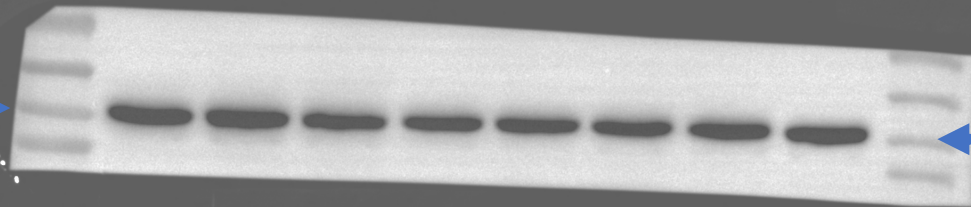

40kDa

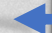

Supplement: Supplementary file 1 — Data S1. [file CNS-30-e14681-s001.zip › unedited b-actin blot for Figure 1.pdf]

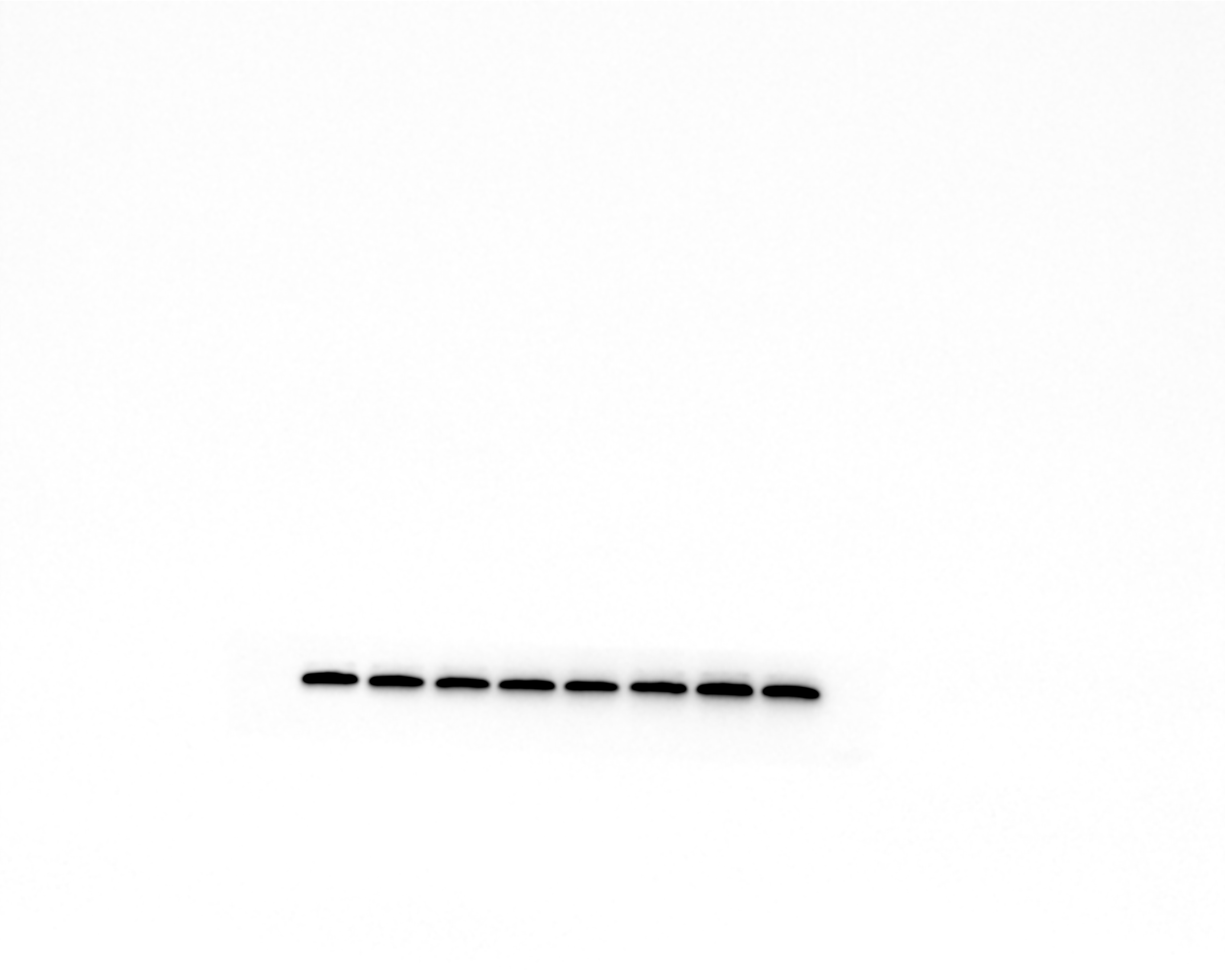

Supplement: Supplementary file 1 — Data S1. [file CNS-30-e14681-s001.zip › unedited b-actin blot for Figure 1.png]

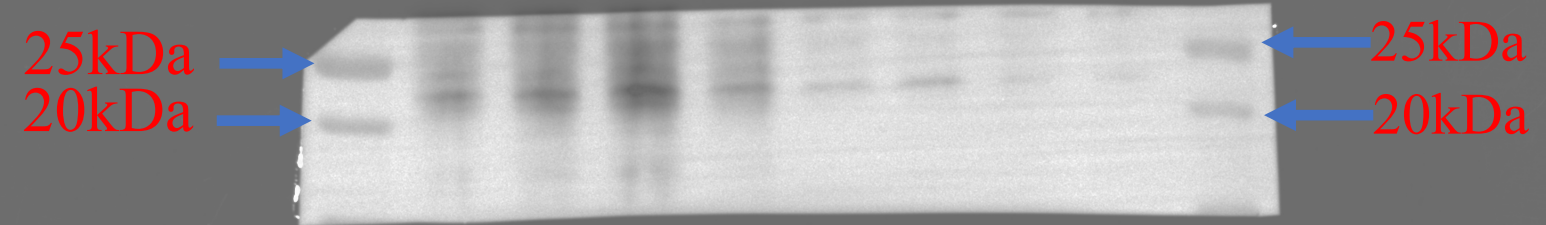

Supplement: Supplementary file 1 — Data S1. [file CNS-30-e14681-s001.zip › unedited prx2 blot for Figure 1.pdf]

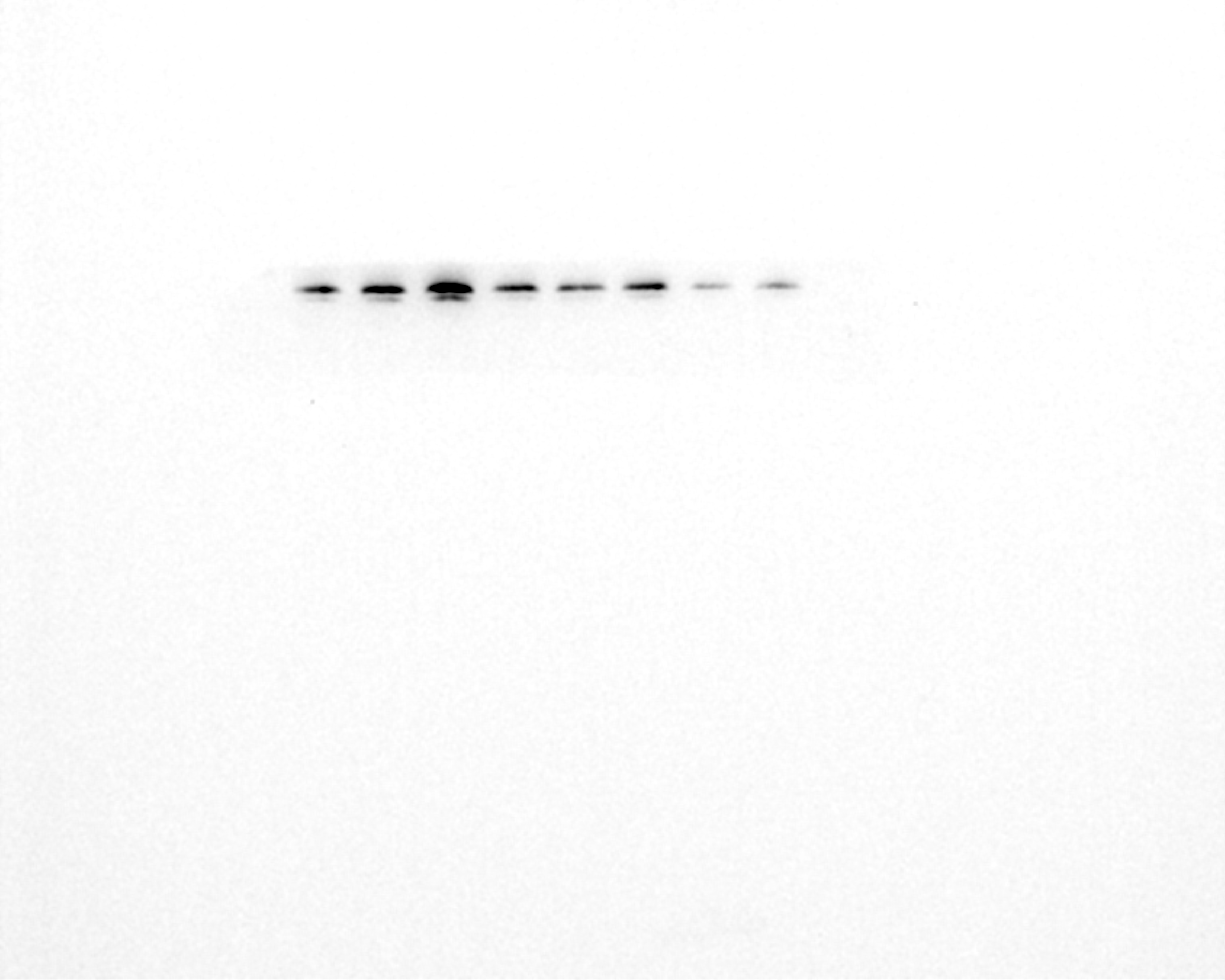

Supplement: Supplementary file 1 — Data S1. [file CNS-30-e14681-s001.zip › unedited prx2 blot for Figure 1.png]
